# Evaluation of respiratory syncytial virus group A and B genotypes among nosocomial and community-acquired pediatric infections in southern Brazil

**DOI:** 10.1186/1743-422X-11-36

**Published:** 2014-02-24

**Authors:** Fernanda de-Paris, Caroline Beck, Luciana de Souza Nunes, Alice Beatriz Mombach Pinheiro Machado, Rodrigo Minuto Paiva, Denise da Silva Menezes, Márcia Rosane Pires, Rodrigo Pires dos Santos, Ricardo de Souza Kuchenbecker, Afonso Luis Barth

**Affiliations:** 1 Universidade Federal do Rio Grande do Sul (UFRGS), Faculdade de Medicina, Programa de Pós-graduação em Ciências Médicas, Rua Ramiro Barcelos 2400, Porto Alegre, RS, Brazil; 2 Instituto de Avaliação de Tecnologia em Saúde/IATS/CNPq, Universidade Federal do Rio Grande do Sul (UFRGS), Faculdade de Medicina, Programa de Pós-graduação em Epidemiologia, Rua Ramiro Barcelos 2400, Porto Alegre, RS, Brazil; 3 Hospital de Clínicas de Porto Alegre (HCPA), Serviço de Patologia Clínica, Unidade de Pesquisa Biomédica–Laboratório de Biologia Molecular, Unidade de Microbiologia e Biologia Molecular, Rua Ramiro Barcelos 2350, Porto Alegre, RS, Brazil; 4 Hospital de Clinicas de Porto Alegre (HCPA), Comissão de Controle de Infecção Hospitalar, Rua Ramiro Barcelos 2350, Porto Alegre, RS, Brazil

**Keywords:** Respiratory syncytial virus, Nosocomial infection, G-protein, Genetic variability, Molecular epidemiology

## Abstract

**Background:**

Respiratory syncytial virus (RSV) is the main cause of lower respiratory tract illness in children worldwide. Molecular analyses show two distinct RSV groups (A and B) that comprise different genotypes. This variability contributes to the capacity of RSV to cause yearly outbreaks. These RSV genotypes circulate within the community and within hospital wards. RSV is currently the leading cause of nosocomial respiratory tract infections in pediatric populations. The aim of this study was to evaluate the G protein gene diversity of RSV amplicons.

**Methods:**

Nasopharyngeal aspirate samples were collected from children with nosocomial or community-acquired infections. Sixty-three RSV samples (21 nosocomial and 42 community-acquired) were evaluated and classified as RSV-A or RSV-B by real-time PCR. Sequencing of the second variable region of the G protein gene was performed to establish RSV phylogenetics.

**Results:**

We observed co-circulation of RSV-A and RSV-B, with RSV-A as the predominant group. All nosocomial and community-acquired RSV-A samples were from the same phylogenetic group, comprising the NA1 genotype, and all RSV-B samples (nosocomial and community-acquired) were of the BA4 genotype. Therefore, in both RSV groups (nosocomial and community-acquired), the isolates belonged to only one genotype in circulation.

**Conclusions:**

This is the first study to describe circulation of the NA1 RSV genotype in Brazil. Furthermore, this study showed that the BA4 genotype remains in circulation. Deciphering worldwide RSV genetic variability will aid vaccine design and development.

## Background

The respiratory syncytial virus (RSV) is considered the leading cause of lower respiratory tract infections in children worldwide [1,2]. RSV also infects adults and is a major pathogen in older adults and immunocompromised individuals [3]. RSV strains are separated into two major groups (A and B) on the basis of antigenic and genetic variability. The main differences are found in the attachment glycoprotein G. RSV G protein interacts with host cell receptors, is a target for neutralizing antibodies, and is highly variable [4]. G protein variability is greater than that of other RSV proteins, both between and within the RSV-A and B groups. Molecular analysis of the second variable region in the G protein has been used to characterize RSV genotypes [5,6]. This variability might contribute to the ability of the virus to cause yearly outbreaks [6,7].

RSV exhibits clear seasonality. Outbreaks occur in the late fall and winter in temperate regions, and during extended periods related to rainy seasons in tropical regions [8-10]. RSV-A and B genotypes show complex fluctuating dynamics. They may co-circulate during a given season, with one or two dominant genotypes that are then replaced in consecutive years [11-13]. These genotypes circulate within the community and within hospital wards. Consequently, nosocomial infection by RSV is observed every year. RSV is the leading cause of nosocomial respiratory infections in pediatric populations. This virus is a particular hazard for premature infants, infants with congenital heart disease or bronchopulmonary dysplasia, and infants and children that are immunodeficient [14,15]. Nosocomial RSV outbreaks can cause severe morbidity and mortality in infants, and carry a substantial financial burden [16-18]. Palivizumab is a humanized monoclonal antibody that is currently recommended as prophylaxis for certain patient groups at risk of severe RSV infection [18]. However, it is too expensive for wide-range use in developing countries such as Brazil [19].

Despite its pathogenic importance, there is no effective vaccine against RSV [20]. Future development of vaccines or pharmacotherapy against RSV requires further understanding of the genetic composition of viral strains prevalent in a target population. The aim of the present study was to evaluate the genetic diversity of the G protein gene in RSV samples collected from young children with nosocomial or community-acquired infections in Southern Brazil.

## Results

Real-time PCR (RT-PCR) results confirmed the presence of both RSV-A and RSV-B groups in the samples (21 from nosocomial RSV patients and 42 from community-acquired RSV patients). RSV-A was the predominant group in both nosocomial and community-acquired samples. Three cases of RSV-A/B co-infection were detected, in two nosocomial samples and one community-acquired sample. Patients with nosocomial RSV infections were older and had longer hospital stays (Table 1).

**Table 1:** Characteristics of patients with hospital-and community-acquired RSV infections treated at Hospital de Clínicas de Porto Alegre, 2010

**Characteristics**	**Patients**		**p-value**
	**Hospital-acquired infection (n = 21)**	**Community-acquired infection (n = 42)**	
Mean age, months [range]	15 [2-94]	9 [3-61]	0.013*
Mean length of stay, days [range]	26.09 [2-73]	10.28 [0.6-71]	0.015*
Deaths	3	1	0.104
RSV-A infection	15	30	
RSV-B infection	8	13	
Mixed RSV-A/B infection	2	1	

Age and length of stay were analyzed using the Mann-Whitney *U* test, and number of deaths, using Fisher’s exact test.

*p-value <0.05.

We were able to obtain 56 sequences (40 RSV-A and 16 RSV-B) for phylogenetic analyses. Seven samples showed no amplification or low-quality sequencing. Samples without amplification may have degraded RNA, and those with low-quality sequencing could be affected by non-specific amplifications. Consequently, these samples were not considered for the phylogenetic analysis.

**Figure 1:**
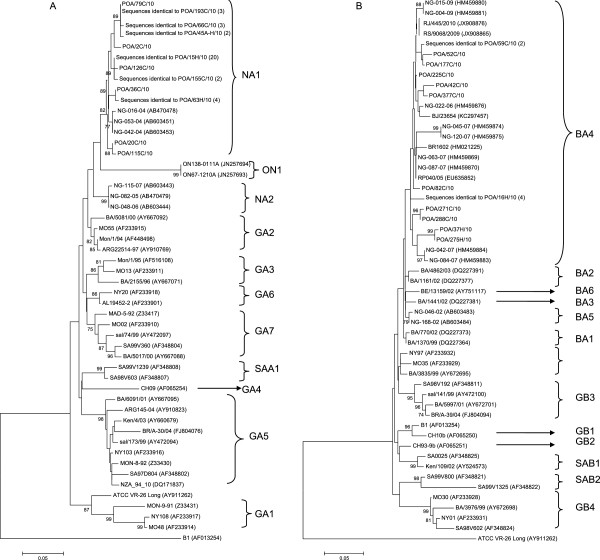
**Phylogenetic trees for RSV-A (A) and RSV-B (B) nucleotide sequences based on the second variable region of the G protein, using the neighbor-joining method and MEGA version 5.05.** Reference strains (AF013254 for group A and AY911262 for group B) were used as outgroup sequences in the tree. Scale bars show that the proportion of nucleotide substitutions and numbers at each branch are bootstrap values determined from 1,000 iterations. Only bootstrap values with > 70% significance are shown. In phylogenetic tree for RSV-A **(A)**–sequences identical to POA/193C/10 were POA/336H/10 and POA/344H/10; sequences identical to POA/66C/10 were POA/335C/10 and POA/312H/10; sequence identical to POA/45A-H/10 was POA/96H/10; sequences identical to POA/15H/10 were POA/169H/10, POA187C/10, POA/166H/10, POA144C/10, POA/136C/10, POA/124C/10, POA/106C/10, POA/62C/10, POA/18C/10, POA/1C/10, POA/203H/10, POA/229C/10, POA/254H/10, POA/278C/10, POA/300C/10, POA/316C/10, POA/354C/10, POA/359H/10 and POA/33H/10; sequence identical to POA/155C/10 was POA171C/10; sequences identical to POA/63H/10 were POA/114H/10, POA/179C/10 and POA/211C/10. In phylogenetic tree for RSV-B **(B)**–sequence identical to POA/59C/10 was POA/121C/10; sequences identical to POA/16H/10 were POA/73H/10, POA/56H/10 and POA/57H/10.

All RSV-A sequences (40 sequences), whether nosocomial or community-acquired, were grouped with the NA1 genotype sequences. This phylogenetic branch, comprising sequences from NA1 isolates and POA samples, presented a bootstrap value of 82% and was clearly distinct from other genotypes (Figure 1). Moreover, the calculated intra-genotype *p*-distance showed a high similarity between RSV-A POA sequences and the NA1 genotype (Table 2).

**Table 2:** **Pairwise nucleotide distances ( *p- *** **distances), intra-genotype and inter-genotype, for RSV-A (NA1) and RSV-B (BA4) isolates**

**Subgroup**	**Genotypes**	**Intra-genotype**	**Inter-genotype (neighbor cluster)**
RSV-A	NA1	0.013	0.053 (NA1 × NA2)
RSV-B	BA4	0.037	0.040 (BA4 × BA2)

Sixteen RSV-B sequences (nosocomial and community-acquired) were grouped with the BA4 genotype sequences. This phylogenetic branch, comprising sequences from BA4 isolates and POA samples, is shown in Figure 1. Therefore, all POA isolates had the 60-nucleotide duplication within the G gene originally reported in BA strains [21]. Calculated *p*-distances showed intra-genotype similarity and inter-genotype diversity among the RSV-B sequences (Table 2).

## Discussion

RSV is recognized as the leading cause of respiratory tract illness in children and is a major nosocomial pathogen. We evaluated RSV genetic variability in nasopharyngeal aspirate samples taken from children with nosocomial and community-acquired RSV infections at a large hospital in southern Brazil during 2010. The patients with nosocomial RSV infection were characterized by prolonged hospital stays and increased mortality (Table 1). Increased risk of severe RSV infection is usually associated with clinical conditions such as prematurity, chronic lung disease, congenital heart disease, and immunosuppression [1,14]. These same conditions are common among children with prolonged hospital stays [15].

RSV-A was the predominant group in both nosocomial (15/21 or 71.43%) and community-acquired (30/42 or 71.43%) infections. Only three cases of RSV-A/B co-infection (4.76%) were observed. In previous RSV studies, co-circulation of groups A and B within the same population has been described during epidemic periods, and with different predominance patterns [12,22]. There have been reports of co-circulation of the RSV-A and RSV-B groups in different years, including in Brazilian populations; however, most sequences have belonged to the RSV-A group [23-25]. Shifts in predominance of particular circulating RSV groups within a given time interval have also been described [21,26,27]. These shifts appear to correlate, at least in part, with G-protein gene variability [22]. However, as we collected samples only during one epidemic period for the present study, considerations about epidemiological patterns are restricted.

Only one RSV-A genotype was found circulating within the study sample. It showed high similarities with the previously identified NA1 genotype isolated from Japan [12]. All RSV-A POA sequences (nosocomial and community-acquired) were grouped with this genotype in a phylogenetic branch which presented a bootstrap value of 82%. The *p*-distance calculated among these sequences was low, demonstrating homogeneity among samples. This is the first study to describe circulation of the Japanese NA1 genotype in Brazil. One could speculate that the introduction of this genotype to Brazil was probably recent and that it became predominant in the Porto Alegre area. Peret and colleagues have hypothesized that a constant shift in predominant genotypes affects protective immunity, allowing for more efficient virus transmission and pathogenicity. Therefore, new RSV strains can be introduced into a given population, but local factors such as strain-specific immunity will determine which strains will circulate successfully and cause outbreak [5].

We found only one circulating RSV-B group genotype in southern Brazil during 2010: BA4. This genotype has been previously described [21]. The phylogenetic branch grouped many sequences, and the *p*-distance also indicated some variability within this phylogenetic group (*p*-distance = 0.037). However, all RSV-B POA genotypes were grouped with the BA genotypes. The first BA genotype was described in Buenos Aires (Argentina) in 1999, and harbors a 60-nucleotide duplication within the G protein gene [28]. It has also been reported in Europe and Asia [12,29,30]. The rapid and worldwide spread of the BA genotype implies that it may have a selective advantage over other circulating strains [31]. However, there may be mutations elsewhere in the genome that confer more efficient replication when compared with other RSV-B genotypes. Indeed, RSV (both RSV-A and RSV-B groups) exhibits rapid evolutionary rates [21], which might justify the emergence of many different genotypes and variability within genotypes as well. The BA4 genotype has circulated worldwide for years and exhibits a high mutation rate; therefore, many BA4 variants could be generated.

In summary, this study evaluated the genetic diversity of RSV within a patient cohort. To our knowledge, this is the first time that circulation of the RSV-A NA1 genotype has been confirmed in Brazil. In addition, we report circulation of the BA4 genotype during 2010. A better understanding of RSV molecular epidemiology will be essential for development of vaccines and antiviral pharmacotherapy against RSV infection. Currently, palivizumab is recommended as a preventive measure in high-risk settings. Unfortunately, this agent is too costly for use in developing countries [19]. Therefore, the development of an efficient vaccine should remain a high priority in managing RSV infection.

## Methods

### Patients

Twenty-one pediatric patients were diagnosed with nosocomial RSV infections at Hospital de Clínicas de Porto Alegre, a tertiary referral center in southern Brazil, in 2010. Diagnostic criteria for nosocomially infected patients included absence of respiratory symptoms at the time of hospital admission and detection of RSV by indirect immunofluorescence in nasopharyngeal aspirate samples within 7 days of admission [14]. These 21 patients account for all nosocomial RSV infections that occurred in 2010. Furthermore, nasopharyngeal aspirate samples from 42 pediatric patients with community-acquired RSV infection were also analyzed. These patients were admitted to the same ward as the nosocomially infected patients 1 week before or after their admission. Community-acquired RSV patients stayed at the hospital for least 3 days.

All nasopharyngeal aspirate samples were obtained for clinical purposes as part of standard care. The study protocol was approved by the Institutional Review Board of Hospital de Clínicas de Porto Alegre.

### Statistical analysis

Differences between the two groups (patients with hospital-and community-acquired RSV infections) were analyzed using the Mann-Whitney *U* test (age and length of hospital stay) or Fisher’s exact test (number of deaths). The significance level was set at p < 0.05 (Table 1). All statistical analyses were performed in SPSS 14.0.

### Sample handling and nucleotide sequencing

Nasopharyngeal aspirate samples were collected in phosphate-buffered saline (PBS) and frozen at -80°C until RNA extraction. The samples were named according to the city of origin (e.g. Porto Alegre–POA), number, and infection setting (nosocomial, H; community-acquired, C). RNA from a 140-μL aliquot of each sample was extracted with a QIAamp Viral RNA Mini Kit (Qiagen, Valencia, CA, USA), according to the manufacturer’s instructions. RSV-positive samples were classified as RSV-A or RSV-B by group-specific real-time PCR, as described previously [32].

For nucleotide sequencing, RT-PCR was performed on extracted RNA using primers GAAGTGTTCAACTTTGTACC/ CAACTCCATTGTTATTTGCC for RSV-A and AAGATGATTACCATTTTGAAGT/CAACTCCATTGTTATTTGCC for RSV-B, under previously described amplification conditions [5]. We amplified the second variable region of the G gene, including the C-terminal region of protein G. PCR products were purified using a PureLink PCR Purification kit (Invitrogen, Carlsbad, CA, USA). Both strands were sequenced in an ABI 3500 Genetic Analyzer (Applied Biosystems, Foster City, CA, USA) using the BigDye Terminator v3.1 Cycle Sequencing Kit (Applied Biosystems, Foster City, CA, USA).

### Phylogenetic analyses

Sequences were assembled using BioNumerics software version 6.0 and aligned with ClustalW. All RSV sequences analyzed covering fragments beginning on nucleotide 615 until the end of the G gene. Phylogenetic analyses were performed with MEGA 5.05 software [33], using the neighbor-joining method and bootstrap analysis (1,000 replicates). Reference sequences for RSV-A and RSV-B strains originating from the United States [6], Uruguay and Argentina [21,34-36], Brazil [23-25], South Africa [37], Kenya [26], New Zealand [38], Japan [7,12,39], Canada [40], China [41] and Belgium [29] were obtained from GenBank.

Pairwise nucleotide distances (*p-*distances), the number of pairwise nucleotide differences divided by the total number of nucleotides in the sequenced segment, were calculated using MEGA 5.05 [33]. A lower *p-*distance indicated a greater similarity in compared sequences. To improve the distribution and appearance of the phylogenetic tree, only unique sequences are shown (Figure 1).

### Nucleotide sequence accession numbers

The GenBank accession numbers of the nucleotide sequences obtained in the present study are: JX524454 to JX524456; JX568889 to JX568900; JX627410 to JX627433; and JX678714 to JX678729.

## Abbreviations

RSV: Respiratory syncytial virus; RT-PCR: Reverse transcriptase polymerase chain reaction; PBS: Phosphate-buffered saline.

## Competing interests

The authors declare that they have no competing interests.

## Authors’ contributions

FP–collected samples and data, ran RT-PCR reactions, evaluated sequences, and drafted the manuscript. CB–collected samples and data and ran RT-PCR and sequencing reactions. LSN–evaluated sequences and constructed the figure and tables. ABMPM, RMP and DSM–collected samples and data for analysis. MRP, RPS and RSK–provided clinical data and support. ALB and RSK–supervised the study and critically revised the manuscript. All authors read and approved the final manuscript.
